# Moderate Hypoxia Induces β-Cell Dysfunction with HIF-1–Independent Gene Expression Changes

**DOI:** 10.1371/journal.pone.0114868

**Published:** 2014-12-12

**Authors:** Yoshifumi Sato, Masahiro Inoue, Tatsuya Yoshizawa, Kazuya Yamagata

**Affiliations:** 1 Department of Medical Biochemistry, Faculty of Life Sciences, Kumamoto University, Kumamoto, Japan; 2 Department of Biochemistry, Osaka Medical Center for Cancer and Cardiovascular Diseases, Osaka, Japan; Niigata University Graduate School of Medical and Dental Sciences, Japan

## Abstract

Pancreatic β-cell failure is central to the development and progression of type 2 diabetes. We recently demonstrated that β-cells become hypoxic under high glucose conditions due to increased oxygen consumption and that the pancreatic islets of diabetic mice but not those of control mice are moderately hypoxic. However, the impact of moderate hypoxia on β-cell number and function is unknown. In the present study, moderate hypoxia induced a hypoxic response in MIN6 cells, as evidenced by increased levels of HIF-1α protein and target genes. Under these conditions, a selective downregulation of *Mafa*, *Pdx1*, *Slc2a2*, *Ndufa5*, *Kcnj11*, *Ins1*, *Wfs1*, *Foxa2*, and *Neurod1*, which play important roles in β-cells, was also observed in both MIN6 cells and isolated pancreatic islets. Consistent with the altered expression of these genes, abnormal insulin secretion was detected in hypoxic MIN6 cells. Most of the hypoxia-induced gene downregulation in MIN6 cells was not affected by the suppression of HIF-1α, suggesting a HIF-1–independent mechanism. Moderate hypoxia also induced apoptosis in MIN6 cells. These results suggest that hypoxia is a novel stressor of β-cells and that hypoxic stress may play a role in the deterioration of β-cell function.

## Introduction

Type 2 diabetes mellitus is a chronic metabolic disease characterized by high blood glucose levels. Pancreatic β-cells sense glucose and secrete appropriate amounts of insulin, which plays a key role in maintaining normal glucose levels. Pancreatic β-cell failure is central to the development and progression of type 2 diabetes. Although the mechanisms underlying β-cell dysfunction are not fully understood, oxidative stress, endoplasmic reticulum (ER) stress, and inflammatory stress are considered to be involved in β-cell failure [1], [2]. Since β-cells require large amounts of oxygen to produce ATP for insulin secretion, adequate oxygen availability is crucial for β-cells. Indeed, we and others recently demonstrated that accelerated mitochondrial function under high glucose conditions increases oxygen consumption, decreases oxygen availability, and renders β-cells hypoxic [3], [4].

When oxygen availability is reduced, hypoxia-inducible factor (HIF)-1 regulates the expression of genes mediating adaptive responses to hypoxia [5]. HIF-1 is composed of an oxygen-sensitive HIF-1α subunit and a constitutively expressed HIF-1β subunit. Under well-oxygenated conditions, HIF-1α is bound by von Hippel-Lindau (VHL) protein. VHL recruits an E3 ubiquitin ligase that targets HIF-1α for proteasomal degradation. Decreased oxygen tension results in reduced hydroxylation, enabling HIF-1α to escape VHL capture and proteasomal degradation. Under hypoxic conditions, HIF-1α forms a heterodimer with HIF-1β to act as a transcription factor, leading to the activation of various target genes. Recent studies revealed that HIF-1 activation by conditional inactivation of the *Vhl* gene in β-cells impairs insulin secretion and glucose homeostasis in mice, indicating the important roles of HIF-1 in β-cells [6], [7], [8]. Although HIF plays an important role, HIF-independent regulation in response to hypoxia is also reported [9]. Hypoxia may play a wider range of roles in deterioration of β-cell function independent of HIF-1 activation.

The mean tissue oxygen tension at the surface of normal mouse pancreatic islets is 44.7–45.7 mmHg (equivalent to 6.3%–6.4% oxygen tension) [10], but the oxygen tension in diabetic islets is unknown. Pimonidazole is widely used for the evaluation of hypoxia [11]. It forms adducts with intracellular molecules under hypoxic conditions, and this adduct formation can be assessed by immunohistochemical analysis [when O_2_ partial pressure is below 10 mmHg (equivalent to 1.4% oxygen tension)] [12] or by more sensitive western blotting analysis [3]. We detected pimonidazole adduct formation in pancreatic islets of animal models of diabetes by western blotting but failed to detect the adduct formation by immunohistochemical analysis, suggesting that β-cells become moderately, but not severely, hypoxic (1.4%–6.3%) under diabetic conditions [3]. The MIN6 cell line was established from insulinoma cells [13], and these cells are normally cultured at 20% oxygen tension (hyperoxia) in contrast to normal pancreatic islets, which are exposed to about 6% oxygen tension *in vivo*. Thus, it is unclear whether the same oxygen tension induces similar responses in both normal islets *in vivo* and MIN6 cells *in vitro*. We reported that hypoxic responses occur at 5%–7% oxygen tension in MIN6 cells [3]. Furthermore, 3% hypoxia for 24 h markedly increased MIN6 cell death (see below). Although the hypoxic conditions in MIN6 cells reflecting the hypoxia in diabetic β-cells are not yet known, 3% oxygen tension may be too severe to reflect an *in vivo* circumstance. Thus, we mostly used 5% O_2_ to induce moderate hypoxia in the present study.

We demonstrated that moderate hypoxia induced downregulation of several β-cell genes, such as *Mafa*, *Pdx1*, *Foxa2*, *Wfs1*, and *Slc2a2*, which play important roles in β-cells. The hypoxia-induced gene downregulation occurred mainly in a HIF-1–independent manner. We also found that moderate hypoxia induces apoptosis in MIN6 cells. These results suggest that moderate hypoxia is a novel stressor of β-cells and that hypoxic stress may play a role in the deterioration of β-cell function.

## Materials and Methods

### Cell culture

MIN6 cells [13] were a gift from Jun-ichi Miyazaki (Osaka University). MIN6 cells were maintained in Dulbecco's modified Eagle's medium (DMEM) containing 25 mM glucose, 10% (v/v) fetal bovine serum, 0.1% (v/v) penicillin/streptomycin, and 50 µM β-mercaptoethanol at 37°C in 5% CO_2_, 95% air. Sustained hypoxic incubation was achieved using a multi-gas incubator (APM-300; ASTEC, Fukuoka, Japan) and an INVIVO_2_400 hypoxia workstation apparatus (Ruskin Technology).

### Animals

C57BL6J mice (KBT Oriental Co., Ltd.) were maintained at the Center for Animal Resources and Development (CARD) of Kumamoto University and were carefully handled in compliance with the animal care guidelines of Kumamoto University. Mice were raised under specific-pathogen free conditions in a 12-h light (7:00–19:00)/12-h dark (19:00–7:00) cycle with free access to water and normal mouse chow (CE-2; CLEA, Tokyo, Japan). Room temperature was stably maintained at 22±1–2°C. Mice were sacrificed by cervical dislocation. This study was approved by the animal research committee of Kumamoto University (Permission Number: B25-105) and all experimental protocols were approved by the Kumamoto University Ethics Review Committee for Animal Experimentation.

### Isolation of pancreatic islets

Pancreatic islets were isolated from C57BL6J mice (18–20 weeks old) by collagenase digestion as described previously [14]. Briefly, after bile duct cannulation and digestion of the pancreas using collagenase (Nitta Gelatin, Osaka, Japan), isolated islets were hand-picked and collected. They were then cultured in RPMI1640 medium supplemented with 11 mM glucose, 10% (v/v) fetal bovine serum, 0.1% (v/v) penicillin/streptomycin, 50 µM β-mercaptoethanol, 10 mM HEPES (Nacalai Tesque, Kyoto, Japan), and 1 mM sodium pyruvate (Nacalai Tesque) at 37°C in 5% CO_2_, 95% air.

### Quantitative real-time RT-PCR

Total RNA from MIN6 cells was extracted by using Sepasol RNA I Super reagent (Nacalai Tesque). Murine islet RNA was extracted using an RNeasy Micro Kit (Qiagen, Tokyo, Japan). cDNA synthesis was then achieved with a PrimeScript RT reagent kit and gDNA Eraser (RR047A; TaKaRa Bio Inc., Shiga, Japan) according to the manufacturer's instructions. Quantitative real-time PCR (qPCR) was performed using SYBR Premix Ex Taq II (RR820A; TaKaRa) in an ABI 7300 thermal cycler (Applied Biosystems, Foster City, CA). The mRNA value of each gene was normalized to that of *TATA-binding protein (Tbp)* or that of *Actb*. The specific primers used are shown in S1 Table.

### Western blotting

MIN6 cells were dissolved in RIPA buffer [50 mM Tris-HCl (pH 8.0), 150 mM NaCl, 0.1% SDS, 1% Nonidet P-40, 5 mM EDTA, 0.5% sodium deoxycholate, 20 mg/ml Na_3_VO_4_, 10 mM NaF, 1 mM PMSF, 2 mM DTT, and protease inhibitor cocktail (1/100)]. Whole-cell lysates were separated by SDS-polyacrylamide gel electrophoresis, transferred to a polyvinylidene fluoride (PVDF) membrane (Immobilon-P; Millipore, Bedford, MA), and probed with primary antibodies. The antibodies against HIF-1α (NB100-479; Novus Biologicals, Littleton, CO), caspase 3 (#9662; Cell Signaling), cleaved-caspase 3 (#9661; Cell Signaling), PARP/cleaved-PARP (#551025; BD Pharmingen), CHOP/GADD153 (L63F7) (#2895; Cell Signaling), and β-actin (A5060; Sigma) were used for specific detection. After incubation with secondary antibodies, the signals were detected by using Chemi-Lumi One Super (Nacalai Tesque).

### ATP measurement

MIN6 cells were seeded at 1×10^5^ cells/well in a 24-well plate. After incubation under hypoxic conditions (5% O_2_) for 30 h in 25 mM glucose containing DMEM, the cells were recovered. CellTiter-Glo Luminescent Cell Viability Assay (Promega Luminescence Assay #G7571) was used according to the manufacturer's instructions. After being washed with PBS, cells were mixed with 100 mM Tris-HCl buffer (pH 7.4) and subsequent CellTiter-Glo buffer. Then, after incubation for 10 min at room temperature, the cells were vigorously resuspended and subjected to a luminofluorescence assay using a GLOMAX 20/20 luminometer system (Promega). ATP concentration was calculated with a standard curve and the cellular ATP content was standardized by cell number.

### Lactate assay

MIN6 cells were seeded at 2×10^6^ cells/well in a 6-well plate. After incubation under hypoxic conditions (5% O_2_) for 30 h in 25 mM glucose containing DMEM, the cells were recovered. An L-lactic acid kit (Roche, Germany) was used according to the manufacturer's instructions. Recovered culture medium was mixed with iced 1.2 M HClO_4_ at the ratio of 1∶1 and cooled on ice for 10 min. After centrifugation at 10,000 rpm at 4°C for 5 min to remove the pellet, the supernatant was neutralized with 1 N KOH and cooled on ice for another 15 min. After further centrifugation in the same fashion, the supernatant was subjected to subsequent enzymatic reactions. The increase in NADH, an end product in this reaction, was determined by means of its light absorbance at 355 nm using a fluorometric microplate reader (FilterMax F5 Multi-Mode Microplate Reader; Molecular Devices, Sunnyvale, CA).

### Determination of mitochondrial complex I activity

A Complex I Enzyme Activity Microplate Assay kit (ab109721; Abcam) was used for specific measurement of mitochondrial complex I activity. MIN6 cells were seeded at 2×10^6^ cells/well in a 6-well plate. After incubation under hypoxic conditions (5% O_2_) for 30 h in 25 mM glucose containing DMEM, scraped cells were sonicated for 20 seconds using a Bioruptor sonicator (COSMO Bio. Co., Ltd.) in a homogenization buffer (10 mM Tris-HCl, pH 6.7, 10 mM KCl, 0.15 mM MgCl_2_, 1 mM PMSF, and 1 mM DTT). The supernatant was recovered as a crude whole-cell extract and subjected to the subsequent activity assay according to the manufacturer's instructions.

### Insulin secretion and insulin content

MIN6 cells were seeded in a 24-well plate at 5×10^5^ cells/well and maintained for 2–3 days. To measure glucose-stimulated insulin secretion under hypoxia (5% O_2_), all buffers and solutions were equilibrated to 5% O_2_ and all procedures were performed in a 5% O_2_-controlled INVIVO_2_400 hypoxia workstation apparatus (Ruskin Technology). MIN6 cells were pre-incubated for 1 h in pH 7.4 Krebs-Ringer-bicarbonate HEPES (KRBH) buffer (120 mM NaCl, 4.7 mM KCl, 1.2 mM KH_2_PO_4_, 2.4 mM CaCl_2_, 1.2 mM MgCl_2_, 20 mM NaHCO_3_, and 10 mM HEPES) containing 2.2 mM glucose and 0.5% (v/v) bovine serum albumin. MIN6 cells were incubated in 2.2 mM or 22 mM glucose containing KRBH buffer for 1 h, and the culture supernatant was recovered to measure insulin secretion. Secreted insulin was standardized by whole-cell protein content [15]. For measurement of insulin content, after being seeded in a 6-well plate at 5×10^4^ cells/well and incubated for 3 days, MIN6 cells were collected by scraping and centrifugation at 5,000 rpm for 5 min. The cell pellet was resuspended in acid-ethanol (1.5% HCl in 70% EtOH) and then rotated overnight at 4°C. After centrifugation at 14,000 rpm for 10 min at 4°C, acid-ethanol extracts were neutralized with 1 M Tri-HCl (pH 7.5) (1∶1). Insulin content was standardized by cell number. Insulin concentration was determined by a MESACUP Insulin ELISA (MBL, Nagoya, Japan) or a mouse insulin ELISA (AKRIN-011T; Shibayagi, Japan) kit, according to the manufacturer's protocol.

### Retrovirus infection

For knockdown of HIF-1α, oligonucleotide encoding shRNA (target sequence: 5′-AGATGAGTTCTGAACGTCG-3′) was cloned into pSUPER.retro vector (Oligoengine). Oligonucleotide encoding CHOP shRNA (target sequence: 5′-GATTCCAGTCAGAGTTCTATG-3′) was cloned into a pSIREN-RetroQ expression vector (Clontech). After pSUPER.retro-HIF-1α, pSUPER.retro-control, pSIREN-RetroQ-CHOP, or pSIREN-RetroQ-control vector was transfected into Plat-E cells, which are a retroviral packaging cell line, retrovirus-containing medium was mixed with polybrene and added to MIN6 cells for infection. Selection was achieved by using puromycin treatment (5 µg/ml) [16], [17].

### Apoptosis assay

After MIN6 cells were incubated under various hypoxic conditions for 24 h, cells were trypsinized and collected by centrifugation at 6,000 rpm for 10 min. An annexin V-FITC apoptosis detection kit (BioVision Research Products, Mountain View, CA) was used according to the manufacturer's instructions. Cells were stained with annexin V-FITC antibody and propidium iodide (PI) for 5 min at room temperature in the dark, and stained cells were immediately analyzed using a FACSCalibur flow cytometer (BD Biosciences) and FlowJo software (Tomy Digital Biology, Tokyo, Japan) [18].

### Statistical analysis

Statistical analysis was performed using Statview J-5.0 software (SAS Institute, Cary, NC). The significance of the differences was assessed with an unpaired t-test and a value of p<0.05 was considered to be statistically significant.

## Results

### The regulation of glucose metabolism in MIN6 cells by hypoxia

**Figure 1:**
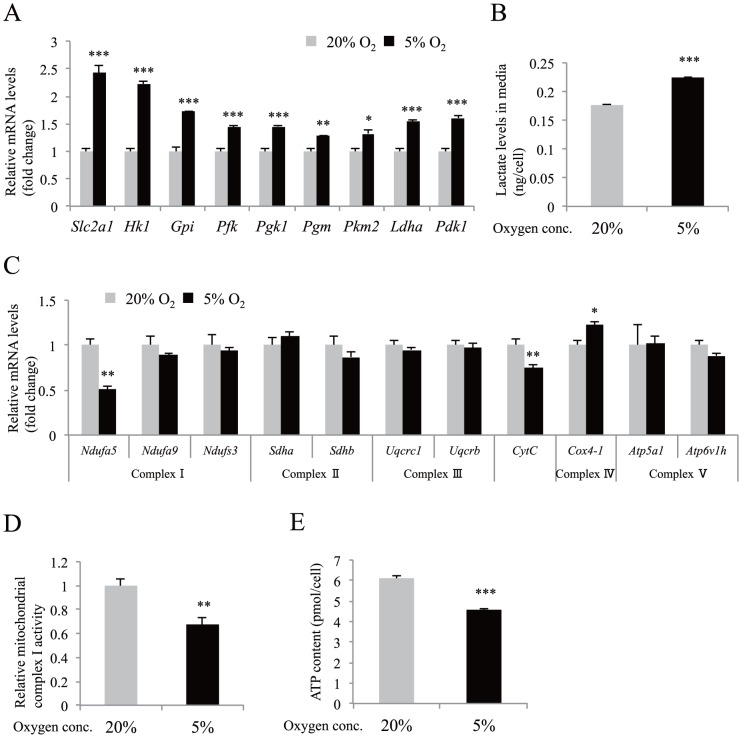
The effect of moderate hypoxia in MIN6 cells. (A) Gene expression analysis by qPCR of known HIF-1 target genes was performed using hypoxic MIN6 cells (n = 4). (B) Lactate concentration in media was measured after MIN6 cells were cultured in hypoxia (n = 4). (C) Gene expression analysis by qPCR of genes encoding mitochondrial respiratory chain complex components (n = 4). (D) Mitochondrial respiratory chain complex I activity under hypoxia was assessed (n = 7). (E) Cellular ATP content under hypoxic conditions was evaluated (n = 4). In all experiments, MIN6 cells were cultured in normoxia (20% O_2_, gray bars) or in moderate hypoxia (5% O_2_, black bars) for 30 h. Each value of mRNA was normalized to that of *TATA-binding protein (Tbp)*. The means ± S.E. (error bars) of values from each group are shown. *, p<0.05; **, p<0.01; ***, p<0.001. Lactate levels and cellular ATP content were standardized by cell number.

We first examined the expression of HIF-1α in MIN6 cells. As described previously [3], moderate levels of hypoxia (5% O_2_) increased the expression of HIF-1α protein (S1 Figure). HIF-1 activates the transcription of genes encoding glucose transporters, glycolytic enzymes, *Pdk1* (encoding pyruvate dehydrogenase kinase), and *Ldha* (encoding lactate dehydrogenase) [19]. Expression of the HIF-1 target genes was significantly increased in the hypoxic MIN6 cells (Fig. 1A). Consistent with the changes in the gene expression of *Pdk1* and *Ldha*, MIN6 cells under hypoxic conditions secreted more lactate (Fig. 1B).

We also investigated the expressions of genes involved in oxidative phosphorylation. Although the expression of most genes was unchanged or slightly increased, the expression levels of *Ndufa5* [encoding NADH-ubiquinone oxidoreductase 1 α subcomplex subunit 5 (complex I)] (51.3% of control; p<0.01) and *Cytc* (encoding cytochrome c, somatic) (74.7% of control; p<0.01) mRNA were significantly lower in the cells (Fig. 1C). The decrease in the levels of both genes by hypoxia has not been reported. Inactivation of *Ndufa5* gene reduces mitochondrial complex I activity [20]. Indeed, complex I activity was decreased in hypoxic MIN6 cells (Fig. 1D). Consistently, MIN6 cells produced less ATP when the cells were cultured in 5% oxygen tension (Fig. 1E). These results suggest that moderate hypoxia mediates a transition of glucose metabolism from an oxidative to a glycolytic pathway in MIN6 cells.

### Insulin secretion by hypoxic MIN6 cells

**Figure 2:**
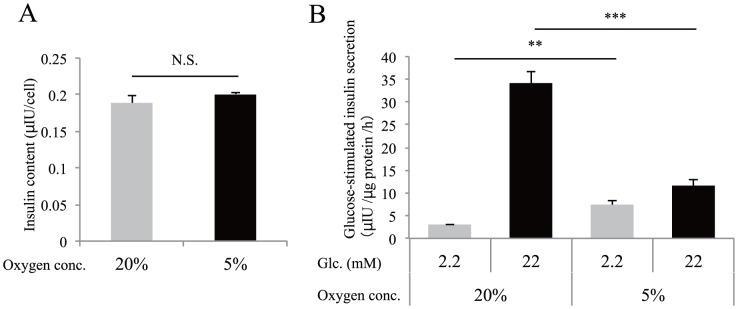
Altered insulin secretion by MIN6 cells under hypoxia. (A) Cellular insulin content was examined after MIN6 cells were incubated in normoxia (20% O_2_, gray bars) or hypoxia (5% O_2_, black bars) for 30 h. Insulin content was standardized by cell number. (B) Glucose-stimulated insulin secretion was examined when MIN6 cells that had been cultured at 20% O_2_ or 5% O_2_ for 40 h were stimulated with low glucose (2.2 mM glucose, gray bars) or high glucose (22 mM glucose, black bars) for 1 h (n = 4). Secreted insulin was normalized to cellular protein levels. Data are shown as the means ± S.E. (error bars) of values from each group. **, p<0.01; ***, p<0.001. N.S., not significant.

We investigated insulin secretion in MIN6 cells under 5% O_2_ tension. Insulin content in MIN6 cells was unchanged under either normoxic or hypoxic conditions (Fig. 2A). In response to high glucose stimulation of MIN6 cells, insulin secretion was markedly increased under normoxic conditions but only slightly increased under hypoxic conditions (Fig. 2B). Interestingly, in response to low glucose stimulation, insulin secretion of MIN6 cells significantly increased in hypoxia compared to in normoxia (Fig. 2B).

### The effect of hypoxia on β-cell gene expression

**Figure 3:**
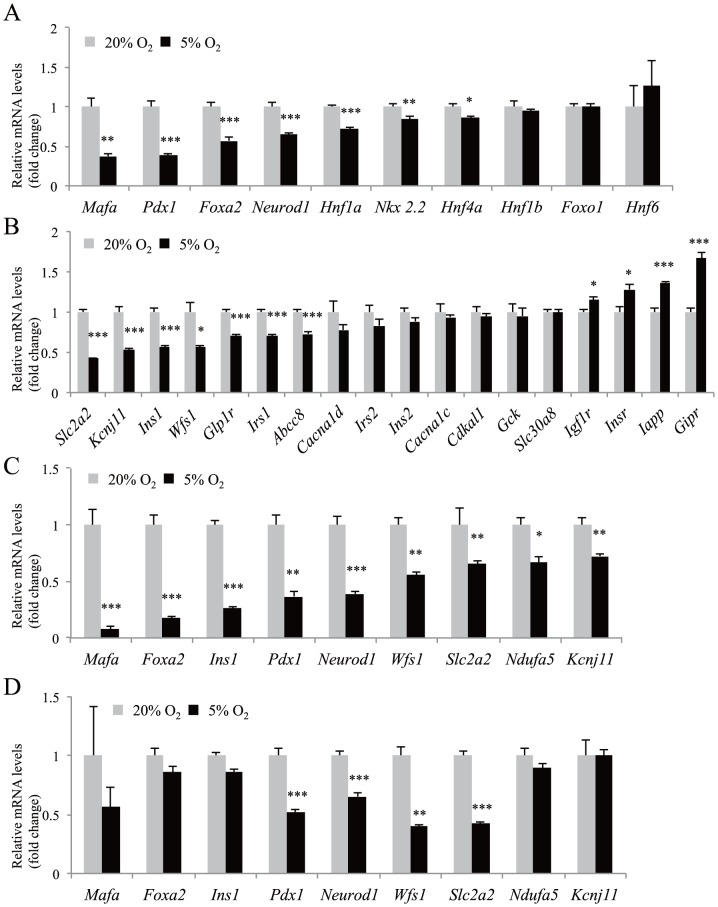
The effect of moderate hypoxia on β-cell gene expression. (A, B) Gene expression analysis by qPCR of β-cell transcription factors (A) and components of the insulin secretion pathway (B) was performed using MIN6 cells incubated in normoxia (20% O_2_, gray bars; n = 4) or in hypoxia (5% O_2_, black bars; n = 4) for 30 h. (C) Gene expression analysis by qPCR was performed when mouse isolated islets were cultured in normoxia (20% O_2_, gray bars; n = 3) or hypoxia (5% O_2_, black bars; n = 3) for 16 h. (D) MIN6 cells were incubated in normoxia (20% O_2_, gray bars; n = 4) or in hypoxia (5% O_2_, black bars; n = 4) for 16 h. The mRNA value of each gene was normalized to that of *Tbp*. The means ± S.E. (error bars) of values from each group are shown. *, p<0.05; **, p<0.01; ***, p<0.001.

We next examined the gene expressions of transcription factors associated with β-cell number and function. Marked downregulation of *Mafa* (37.3% of control; p<0.01), *Pdx1* (38.6% of control; p<0.001), and *Foxa2* (57.2% of control; p<0.001) genes was detected following 5% oxygen tension for 30 h in MIN6 cells (Fig. 3A). Expression of *Neurod1*, *Hnf1a*, *Nkx2.2*, and *Hnf4a* mRNA was also significantly decreased (Fig. 3A). FoxO1 is implicated in the prevention of β-cell dedifferentiation [21]. The expression of *Foxo1* mRNA was unchanged. Activation of Sox9 in β-cells leads to a downregulation of β-cell genes, including *Pdx1* [22]. *Sox9* gene expression was not detected under both 20% and 5% O_2_ conditions (data not shown).

These transcription factors play important roles in β-cells through the regulation of their target genes. We then examined the expression of components of the insulin secretion pathway. There was a significant reduction in *Slc2a2* (encoding GLUT2), *Kcnj11* (encoding Kir6.2), *Ins1*, *Wfs1*, *Glp1r*, *Irs1*, and *Abcc8* (encoding SUR1) mRNA expression in MIN6 cells under hypoxia (Fig. 3B). Islet amyloid polypeptide (IAPP) aggregates into oligomers and form fibrils in the islets, and the amyloid deposits are associated with reduced β-cell mass in type 2 diabetic patients [23]. An increased *Iapp* mRNA level was detected in the hypoxic MIN6 cells. Regarding the receptors, *Glp1r* mRNA was significantly decreased, whereas expression of *Igf1r*, *Insr*, and *Gipr* mRNA was significantly increased under hypoxic conditions (Fig. 3B).

In mice islets under the same hypoxic conditions, we investigated the expression of *Mafa*, *Pdx1*, *Slc2a2*, *Ndufa5*, *Kcnj11*, *Ins1*, *Wfs1*, *Foxa2*, and *Neurod1* genes that exhibited moderate or severe reduction (more than 35%) in MIN6 cells. Hypoxia-induced downregulation of the genes was confirmed in the islets (Fig. 3C). Furthermore, downregulation of *Pdx1*, *Neurod1*, *Wfs1*, and *Slc2a2* genes was detected at an earlier time (16 h) after hypoxic exposure in MIN6 cells (Fig. 3D).

### The role of HIF-1 in hypoxia-induced gene downregulation in MIN6 cells

**Figure 4:**
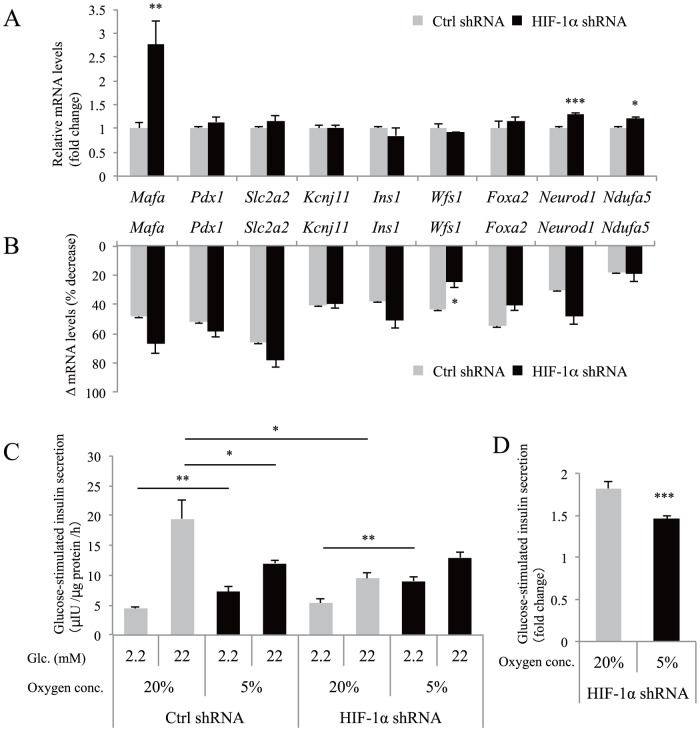
The role of HIF-1 in hypoxia-induced gene downregulation and insulin secretion defects in MIN6 cells. (A) Control (Ctrl) MIN6 cells (gray bars) and HIF-1α knockdown MIN6 cells (black bars) were cultured in normoxia (20% O_2_) and the relative expression levels of β-cell genes were evaluated by qPCR analysis. Each value of mRNA was normalized to that of *Actb*. (B) Downregulation of mRNA levels (Δ mRNA levels) by 5% O_2_. Δ mRNA levels indicate (1-expression levels at 5% O_2_/expression levels at 20% O_2_) ×100 (%). (C) Ctrl MIN6 cells (n = 11) and HIF-1α knockdown MIN6 cells (n = 9) that had been cultured in normoxic (20% O_2_, gray bars) or hypoxic (5% O_2,_ black bars) conditions for 40 h were stimulated with 2.2 mM glucose or 22 mM glucose for 1 h. Secreted insulin was normalized to cellular protein levels. (D) The fold change in glucose-stimulated insulin secretion (insulin level at 22 mM glucose divided by that at 2.2 mM glucose) is indicated (n = 9). Data are shown as the means ± S.E. (error bars) of the values from each group. *, p<0.05; **, p<0.01; ***, p<0.001.

Transgenic mice with β-cell–targeted overexpression of HIF-1 by deletion of the *Vhl* gene exhibited reduced expression of *Pdx1*, *Mafa*, and *Slc2a2* genes when they developed diabetes [22]. We then investigated the role of HIF-1 in hypoxia-induced gene downregulation in MIN6 cells. To this end, we introduced HIF-1α shRNA into MIN6 cells, and successful HIF-1α knockdown was confirmed under hypoxic conditions (S2A Figure). *Slc2a1* gene expression is regulated by HIF-1α [24], and upregulation of *Slc2a1* in hypoxic MIN6 cells was almost completely abolished by knockdown of HIF-1α (S2B Figure). Unexpectedly, knockdown of HIF-1α markedly increased the expression of *Mafa* mRNA under normoxic conditions (Fig. 4A), suggesting that HIF-1 is involved in the regulation of the basal expression levels of the gene. Expression of *Neurod1* and *Ndufa5* was also slightly, but significantly, increased by HIF-1α knockdown. However, knockdown of HIF-1α did not affect the degree of hypoxia-mediated downregulation of *Mafa*, *Pdx1*, *Slc2a2*, *Kcnj11*, *Ins1*, *Wfs1*, *Foxa2*, *Neurod1*, and *Ndufa5*, indicating an HIF-1–independent mechanism (Fig. 4B). In contrast, downregulation of *Wfs1* was partially suppressed, indicating that hypoxia-induced downregulation of *Wfs1* is partially HIF-1 dependent.

We next investigated insulin secretion in HIF-1α knockdown and control MIN6 cells under both 20% and 5% O_2_ conditions (Fig. 4C). As described previously [25], knockdown of HIF-1α in MIN6 cells markedly impaired high glucose-stimulated insulin secretion under normoxic conditions (Fig. 4C). Insulin secretion at 2.2 mM glucose by HIF-1α knockdown MIN6 cells was significantly increased in hypoxia compared with normoxia (Fig. 4C). The fold change in insulin levels by glucose stimulation (insulin level at 22 mM glucose divided by that at 2.2 mM glucose) was significantly decreased in HIF-1α knockdown MIN6 cells under hypoxic conditions (Fig. 4D).

### Moderate hypoxia induces apoptosis in MIN6 cells

**Figure 5:**
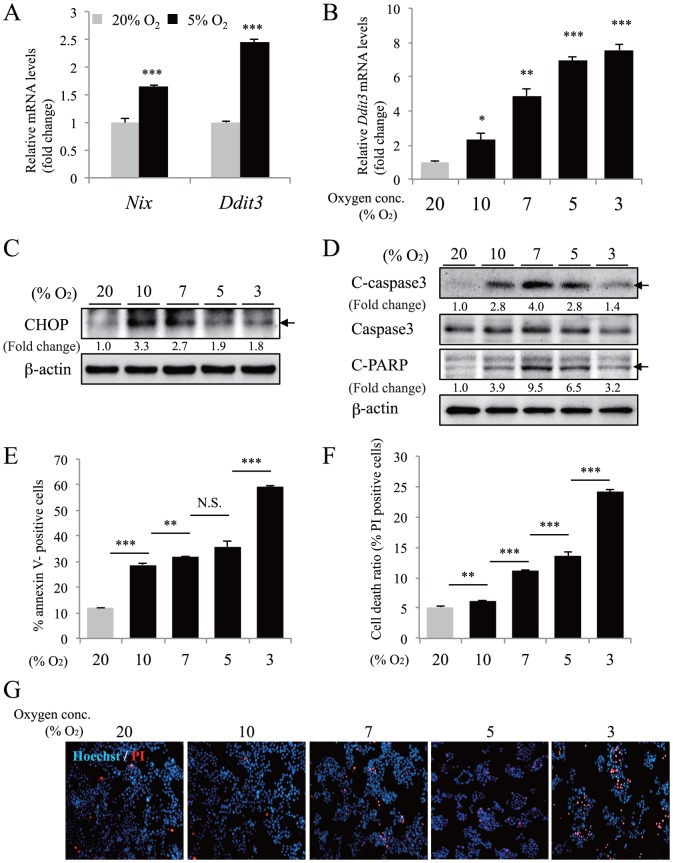
Moderate hypoxia-mediated cell death in MIN6 cells. (A) MIN6 cells were incubated in normoxia (20% O_2_, gray bars) or hypoxia (5% O_2_, black bars) for 30 h and gene expression of *Nix* and *Ddit3* was examined by qPCR (n = 4). (B) MIN6 cells were cultured under various oxygen conditions (3%, 5%, 7%, 10%, and 20% O_2_) for 4 h and *Ddit3* mRNA was examined by qPCR (n = 3). The mRNA value of each gene was normalized to that of *Tbp*. (C) MIN6 cells were cultured in the indicated condition for 3 h and CHOP protein was detected by western blotting. β-actin was used as a loading control. The fold change (band intensity of CHOP divided by band intensity of β-actin) is indicated. (D) Cleaved forms (indicated with arrows) of caspase 3 protein (C-casp3) or PARP protein (C-PARP) were detected by western blotting using MIN6 cells incubated in the indicated condition for 24 h. β-actin was used as a loading control. The fold change (band intensity of C-casp3 and C-PARP divided by band intensity of β-actin) is indicated. (E) The annexin V-positive cell ratio was evaluated by flow cytometric analysis (n = 5) after MIN6 cells under the indicated conditions for 24 h were stained with annexin-FITC antibody. (F) The total cell death ratio was also evaluated by staining MIN6 cells with propidium iodide (PI) (n = 5) after hypoxic culture for 24 h. (G) In the same condition as (F), cells were double-stained with Hoechst 33342 and PI and cell images were captured by using a fluorescence microscopy. Representative images are shown. The data are shown as the means ± S.E. (error bars) of values from each group. *, p<0.05; **, p<0.01; ***, p<0.001.

Nix, a critical mediator of apoptosis, is increased in *Pdx1* haploinsufficient mouse islets [26] and *Pdx1* ^+/−^ islets exhibit apoptosis [27], [28]. NeuroD1-deficient mice show increased levels of apoptosis in pancreatic endocrine cells [29]. In addition, conditional deletion of *Wfs1* in β-cells increases ER stress and apoptosis with elevated expression of CHOP, a pro-apoptotic transcription factor [30], [31]. We detected a significant decrease in *Pdx1*, *Neurod1*, and *Wfs1* gene expression in MIN6 cells under 5% oxygen conditions. Next, we investigated whether moderate hypoxia can induce apoptosis in MIN6 cells. As shown in Fig. 5A, exposure to 5% O_2_ for 30 h induced the upregulation of *Nix* and *Ddit3* (encoding CHOP) genes. An increase in *Ddit3* mRNA and CHOP protein levels was detected as early as 3–4 h following exposure to hypoxia (3%–10% O_2_) (Fig. 5B and 5C). Activation of caspase 3 mediates the induction of apoptosis downstream of CHOP. Moderate hypoxia induced activated (cleaved) caspase 3 protein expression and cleavage of poly (ADP-ribose) polymerase (PARP), a substrate of caspase 3 (Fig. 5D).

Consistent with these results, flow cytometric analysis revealed that moderate hypoxia increased the numbers of annexin V-positive cells and PI-positive dead cells (Fig. 5E and 5F), suggesting the induction of apoptosis in MIN6 cells. Fluorescent staining confirmed increased PI-positive cells in hypoxic MIN6 cells (Fig. 5G). MIN6 cells cultured under 3% O_2_ conditions exhibited reduced expression of C-caspase 3 and C-PARP compared with 5%–10% O_2_ conditions (Fig. 5D), indicating that cell death induced by 3% O_2_ is not typical apoptosis. Thus, the pattern of cell death may depend on the oxygen tension. Absence of caspase activation is a feature of necrotically dying cells [32], [33]. It is therefore possible that exposure to 3% O_2_ for 24 h may have caused necrotic cell death in the MIN6 cells. In contrast to MIN6 cells, an increase in annexin V- and PI-positive cells was not observed when a mouse hepatocellular carcinoma cell line Hepa1-6 was exposed to moderate hypoxia (S3 Figure). Collectively, these findings indicate that moderate hypoxia promotes the induction of apoptosis in MIN6 cells.

### Role of apoptosis in hypoxia-induced gene downregulation in MIN6 cells

**Figure 6:**
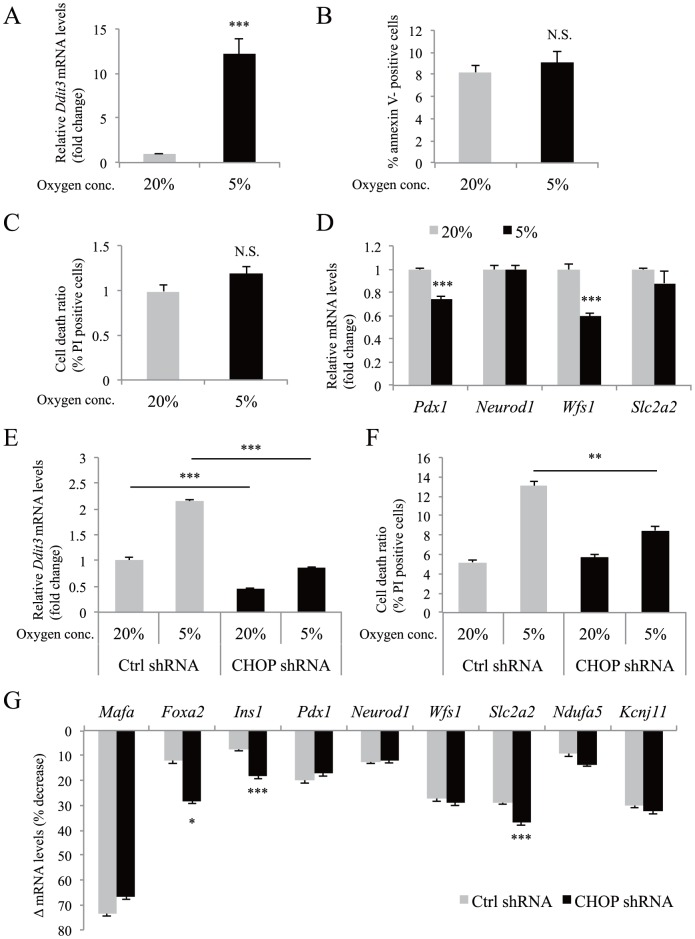
The role of apoptosis in hypoxia-induced gene downregulation in MIN6 cells. (A) MIN6 cells were incubated at 5% O2 for 10 h and the expression levels of *Ddit3* mRNA were examined by qPCR (n = 4). (B, C) Annexin V-positive cell (B) and PI-positive cell death (C) ratios were evaluated by flow cytometric analysis (n = 6) after MIN6 cells were cultured at 5% O2 for 10 h. (D) Expression levels of *Pdx1*, *Neurod1*, *Wfs1*, and *Slc2a2* were examined by qPCR (n = 4) in the same conditions as in (A). (E) Ctrl MIN6 cells (gray bars) and CHOP knockdown MIN6 cells (black bars) were incubated either in normoxia (20% O_2_) or in hypoxia (5% O_2_) for 40 h and the expression levels of *Ddit3* mRNA were examined by qPCR (n = 3). (F) Ctrl MIN6 cells (gray bars) (n = 5) and CHOP knockdown MIN6 cells (black bars) (n = 5) were incubated either in normoxia (20% O_2_) or in hypoxia (5% O_2_). The PI-positive cell death ratio was evaluated by flow cytometric analysis. (G) Ctrl MIN6 cells (gray bars) and CHOP knockdown MIN6 cells (black bars) were incubated in the same conditions as in (E). Downregulation of mRNA levels (Δ mRNA levels) by 5% O_2_. Δ mRNA levels indicate (1-expression levels at 5% O_2_/expression levels at 20% O_2_) ×100 (%). In qPCR analysis, the mRNA value of each gene was normalized to that of *Tbp*. The data are shown as the means ± S.E. (error bars) of values from each group. *, p<0.05; **, p<0.01; ***, p<0.001.

Next, we examined the role of apoptosis in hypoxia-induced gene downregulation. The expression levels of four genes (*Pdx1*, *Neurod1*, *Wfs1*, and *Slc2a2*) that exhibited significant reductions after exposure to 5% oxygen for 16 h (Fig. 3D), were examined at an earlier time point. *Ddit3* gene expression was significantly increased after exposure to 5% oxygen for 10 h (Fig. 6A), but annexin V-positive and PI-positive cells were not increased (Fig. 6B and 6C). Under these conditions, the levels of *Pdx1* and *Wfs1* mRNA were already significantly decreased (Fig. 6D), suggesting that the gene downregulation occurs prior to apoptosis.

Zheng et al. [34] reported the important role of CHOP in hypoxia-mediated apoptosis in MIN6 cells. Therefore, we also examined the gene expression in CHOP knockdown MIN6 cells (Fig. 6E). Suppression of *Ddit3* mRNA by shRNA significantly decreased the number of hypoxia-induced PI-positive cells (Fig. 6F). However, it did not rescue the degree of hypoxia-mediated downregulation of *Mafa*, *Foxa2*, *Ins1*, *Pdx1*, *Neurod1*, *Wfs1*, *Slc2a2*, *Ndufa5*, and *Kcnj11* genes (Fig. 6G). These results suggest that hypoxia-induced downregulation of β-cell genes is unlikely to be a secondary effect of apoptosis.

## Discussion

We recently demonstrated that pimonidazole adducts in islets were remarkably increased in two different model mice of diabetes, KK-Ay and ob/ob, compared with age- and sex-matched control mice [3]. Bensellam et al. [4] detected HIF-1α–positive islet cells in diabetic db/db mice. Furthermore, upregulation of hypoxia-related genes (e.g. *Ldha* and *Vegfa*) is reported in the islets of Zucker Diabetic Fatty (ZDF) rats [35]. Collectively, these results strongly suggest that β-cell hypoxia occurs *in vivo* in the progression of diabetes.

In the present study, we demonstrated that moderate hypoxia leads to the selective downregulation of *Mafa*, *Foxa2*, *Ins1*, *Pdx1*, *Neurod1*, *Wfs1*, *Slc2a2*, *Ndufa5*, and *Kcnj11* genes in both MIN6 cells and mouse islets. Pdx1 and MafA have been reported to control *Slc2a2* gene expression in β-cells [36], [37]. Furthermore, Pdx1, NeuroD1, and MafA strongly and synergistically activate the insulin promoter [38]. In addition, *Kcnj11* and *Abcc8* genes are direct target genes of Foxa2 [39]. Thus, hypoxia-induced downregulation of *Ins1*, *Slc2a2*, and *Kcnj11* mRNA may be explained, at least in part, by the reduced expression of these transcription factors.

Recently, selective gene repression by hypoxia has received increased attention [40], [41]. Our HIF-1α knockdown experiments revealed that the hypoxia-mediated gene downregulation is mainly HIF-1 independent. Hypoxia-induced microRNAs [42] and transcriptional repressors [43] may be involved in the gene repression. Although cellular ATP levels are usually preserved when cells are cultured under the conditions of an O_2_ partial pressure of 10 mmHg or higher [44], ATP levels were significantly decreased in moderately hypoxic MIN6 cells (Fig. 1E). AMP-activated kinase (AMPK) activation leads to decreased levels of mRNAs encoding cyclin A and cyclin B1 by controlling their stability [45]. Decreased ATP-mediated AMPK activation may also be involved in the hypoxia-induced gene downregulation. Further studies are needed to elucidate the mechanism.

Furthermore, we showed that hypoxic MIN6 cells exhibited increased insulin secretion at 2.2 mM glucose compared with normoxic MIN6 cells (Fig. 2B). The activation of HIF-1 in MIN6 cells by hypoxia is a possible mechanism for this observation, because glucose uptake and glycolysis is promoted by HIF-1, even at low glucose concentrations. However, higher insulin secretion at 2.2 mM glucose under hypoxic conditions was also found in HIF-1α knockdown MIN6 cells (Fig. 4C), suggesting the contribution of an HIF-1–independent mechanism. A previous study has shown that the β-cell–specific deletion of the *Foxa2* gene results in increased insulin secretion at lower blood glucose concentrations due to reduced *Kcnj11* and *Abcc8* gene expression [39]. The decreased expression of *Foxa2*, *Kcnj11*, and *Abcc8* (Fig. 3A and 3B) is likely to contribute to the abnormal secretion of insulin at 2.2 mM glucose. In addition, glucose-stimulated insulin secretion was also impaired in hypoxic MIN6 cells (Fig. 2B). Similarly, the upregulation of HIF-1 in MIN6 cells could play a role in the impairment of insulin release in response to high glucose [6], [7], [8]. However, the defect in insulin secretion under high glucose levels was also observed in HIF-1α knockdown MIN6 cells (Fig. 4C). We found an HIF-1–independent reduction of *Pdx1*, *Mafa*, and *Slc2a2* gene expression in hypoxic MIN6 cells (Fig. 4A and 4B), and mice with mutations in these genes exhibit impaired glucose-stimulated insulin secretion [36], [46], [47]. Their reduced expression may also be related to the defect in insulin secretion in MIN6 cells under hypoxic conditions.

Hypoxia induces ER stress-mediated apoptosis in cancer cells or mouse fibroblasts at less than 0.1% O_2_ (severe hypoxia or anoxia) [34]. Indeed, mild hypoxia (5%–7% O_2_) did not induce apoptosis in Hepa1-6 cells (S2 Figure). In contrast, MIN6 cells underwent activation of caspase 3 and apoptosis in response to 5%–7% O_2_, suggesting that β-cells are vulnerable to hypoxia-mediated apoptosis. Apoptosis is believed to constitute the main form of β-cell death in type 2 diabetes [48]. β-cell hypoxia may be one of the causes of the apoptosis.

In conclusion, we found that moderate hypoxia induces downregulation of selective genes and apoptosis in β-cells. Exposure to high glucose induces β-cell damage, which is known as glucotoxicity [49]. High glucose-induced β-cell hypoxia may be involved in the mechanism of glucotoxic damage. Our findings suggest that hypoxia is a novel stressor of β-cells and that hypoxic stress may play a role in the deterioration of β-cell function. A better understanding of hypoxic stress may lead to new therapeutic approaches and agents for type 2 diabetes.

## Supporting Information

S1 Figure **Stabilization of HIF-1α protein in hypoxic MIN6 cells.** When MIN6 cells were cultured in normoxia (20% O_2_) or in moderate hypoxia (5% O_2_) for 30 h, HIF-1α protein was detected by western blotting. β-actin was used as a loading control.
(EPS)

S2 Figure **Expression of HIF-1α protein and** ***Slc2a1*** **mRNA in HIF-1α knockdown MIN6 cells.** (A) Control MIN6 cells (Ctrl) and HIF-1α knockdown MIN6 cells (HIF-1α knockdown) were cultured either in normoxia (20% O_2_) or hypoxia (5% O_2_) for 6 h, and HIF-1α protein was detected by western blotting. β-actin was used as an internal control. (B) Ctrl (gray bars) and HIF-1α knockdown MIN6 cells (black bars) were cultured either in normoxia (20% O_2_) or hypoxia (5% O_2_) for 36 h and the relative expression levels of *Slc2a1* were evaluated by qPCR analysis (n = 3).
(EPS)

S3 Figure **Moderate hypoxia-mediated cell death in Hepa1-6 cells.** (A) The annexin V-positive cell ratio was evaluated by flow cytometric analysis (n = 4) after Hepa1-6 cells under the indicated conditions for 24 h were stained with annexin-FITC antibody. (B) The total cell death ratio was also evaluated by staining of Hepa1-6 cells with propidium iodide (PI) (n = 4) after hypoxic culture for 24 h. Data analysis was performed using Flowjo. The means ± S.E. (error bars) of values from each group are shown. N.S., not significant.
(EPS)

S1 Table **Primer sequences for qPCR analysis.**
(PDF)
