# Supplementary material for: Moderate Hypoxia Induces β-Cell Dysfunction with HIF-1–Independent Gene Expression Changes
Source: PLoS One. 2014 Dec 12;9(12):e114868. doi: 10.1371/journal.pone.0114868 (PMC4264765; doi:10.1371/journal.pone.0114868)
Supplement: S1 Table — Primer sequences for qPCR analysis. (PDF) [file pone.0114868.s004.pdf]

Table S1

Primer sequences for qPCR analysis (1)

Gene name	Forward	Reverse
Slc2a1	GCTTATGGGCTTCTCCAAACT	GGTGACACCTCTCCCACATAC
Hk1	TGCCTCTGGGCTTCACCTTC	CCACACAGTCGGTGGCTTTG
Gpi	CAAGACGCCCCTGGAGAAGA	TCCATGTCACCCTGCTGGAA
Pfk	ATGGCTACCGTGGACCTGGA	CACAGCCCTGACAGCAGCAT
Pgk1	GGGTCGTGATGAGGGTGGAC	CTGGGCCCACACAATCCTTC
Pgm	ATGCTGACAGGCTCGCTGTG	CCGAGCTGTTGGGCTCTGTT
Pkm2	TTGTGCGAGCCTCCAGTC	ACTCCGTGAGAACTATCAAAGC
Ldha	ATGGCAACCCTCAAGGACCA	GAGGCTGCCATGCTGGAGAT
Pdk1	GGACTTCGGGTCAGTGAATGC	TCCTGAGAAGATTGTCGGGGA
Ndufa5	GTCAAGGCGGAGCCAGATGT	TTAGCAGGCGGCTCTTCCAC
Ndufa9	GCAGCCGGTGTATGTTGCAG	AGGTACCGGTTTGGCCCAGT
Ndufs3	CCTGCTGTCTCTGCGGTTCA	TGCCAGCTCCACTGGTTCAG
Sdha	TGTGCGCACTGCAGACCATA	CCTCCAGTGTTCCCCAAACG
Sdhb	CCAGGAGGGCAAGCAACAGT	AGTAGCTGGGGCAGCTGGTG
Uqcrc1	GCGGGAGATGCAGGAAAATG	CCTCCACAGCCTGGGCTAGA
Uqcrb	GGGCCGATCTGCTGTTTCAG	GGTCCAGGGCTCTCTTAATTCG
CytC	TCCATCAGGGTATCCTCTCC	GGAGGCAAGCATAAGACTGG
Cox4-1	TGGGTGGCCATGCAGACCAAGCG	CCGGCAGGCAGCGGGCTCTC
Atp5a1	GGAGGAGACTGGGCGTGTGT	GCATTACCGAGGGCGTCAAC
Atp6v1h	CCGGGGAAAACGGGTTATTG	CCGAGCAGCAGCAGTCTGAG
Mafa	CAGCAGCGGCACATTCTG	GCCCGCCAACTTCTCGTAT
Pdx1	AAGCTCGCTGGGATCACTGG	GGAAGGTGGTGGTGGAGGTG
Foxa2	GCCAGTAGCGGAGGCAAGAA	CACCTCGCTTGTGCTCCTGA
Neurod1	TCCCACGTCTTCCACGTCAA	TTTCAAACTCGGCGGATGGT
Hnf1a	AAGAGCCCACAGGCGATGAG	TGGATGCACTCCGCCCTATT
Nkx2.2	CTTGGTCAGGGACGGCAAAC	GGTGCTGGCCGAGCTGTACT
Hnf4a	GCCACAGTTTTCCACCAAGAG	AAGGAGGACGTCTGCTTCTGA
Hnf1b	CTGGACCCAGGCCACAGTCT	GTAGGGCTGCCAGGCTGCTA
Foxo1	CATCTGCCATGAACCGCTTG	TGGTGGAGGACACCCATCCT
Hnf6	AGTCGGGTCGGGAGACCTTC	TTTGGGGGTGTTGCCTCTGT
Tcf7l2	GGCGCTAACGACGAGCTGAT	CTCTTGGCCGCTTCTTCCAA
Slc2a2	CGTCCTACGGCTCTGGCACT	CACCCCAGCGAAGAGGAAGA
Kcnj11	CTGTCCCGAAAGGGCATTAT	CGTTGCAGTTGCCTTTCTTG
Ins1	ACCCACCCAGGCTTTTGTCA	GGCGGGACTTGGGTGTGTAG
Wfs1	ACGAAGTGCTGGGGAAGCAG	GGGTCTCCAGCCTTGGCTTT

Table S1

Primer sequences for qPCR analysis (2)

Gene name	Forward	Reverse
Glp1r	TGAAGAGCCGCATCTTGCTG	CTCCGTTGAGTGGTGCGATG
Irs1	CCAGCTCCACCCAGCTCCTA	TGCCCAACTCAACTCCACCA
Abcc8	TCAACTTGTCTGGTGGTCAGC	GAGCTGAGAAAGGGTCATCCA
Cacna1d	GGGGTCCAGCTGTTCAAGGGGGAA	GCATGATGAGGACGAACATCATG
Irs2	CAGGAGCAGGAGGGCTGGTA	GGAGAGAGGCGCTGCAAGAG
Ins2	CTGGCCCTGCTCTTCCTCTG	ACAATGCCACGCTTCTGCTG
Cacna1c	CAGGAGGTGATGGAGAAGCCA	CTGCAGGCGGAACCTGTTGTT
Cdkal1	TTGGCACAGATCTCCCCACA	TTGAAGTCGGCCACGCAGTA
Gck	ACAAGGAGGGGAGCCCAGTC	CCCCCACTTTCACCAGCATC
Slc30a8	AGGTGGTGGGTGGACACGTT	AAAAGGCGCTCACAGGCAAG
Igf1r	GGCATGGCCTACCTCAATGC	AGAGGACGACCCCGAAGGAC
Insr	GTGTACGTGCCGGACGAGTG	ACACGGGTCTCTGCCTCACC
Iapp	GCCAGCTGTCCTCCTCATCC	CCGTGTTGCACTTCCGTTTG
Gipr	GATGGCCAGTGGGGATCTTG	CAGCAGCTGATCTCGGGTGA
Chop	ATATCTCATCCCCAGGAAACG	TCTTCCTTGCTCTTCCTCCTC
Tbp	CCCCTTGTACCCTTCACCAAT	GAAGCTGCGGTACAATTCCAG
